# Individual differences in mental imagery in different modalities and levels of intentionality

**DOI:** 10.3758/s13421-021-01209-7

**Published:** 2021-08-30

**Authors:** Georgia A. Floridou, Kaya J. Peerdeman, Rebecca S. Schaefer

**Affiliations:** 1 Health, Medical and Neuropsychology Unit, Leiden University, Leiden, the Netherlands; 2 Department of Music, University of Sheffield, 34 Leavygreave Road, Sheffield, S3 7RD UK; 3 Leiden Institute for Brain and Cognition (LIBC), Leiden, the Netherlands; 4 Academy for Creative and Performing Arts, Leiden University, Leiden, the Netherlands

**Keywords:** Mental imagery, Stimulus modality, Intentionality, Age, Sex, Background experience

## Abstract

Mental imagery is a highly common component of everyday cognitive functioning. While substantial progress is being made in clarifying this fundamental human function, much is still unclear or unknown. A more comprehensive account of mental imagery aspects would be gained by examining individual differences in age, sex, and background experience in an activity and their association with imagery in different modalities and intentionality levels. The current online study combined multiple imagery self-report measures in a sample (*n* = 279) with a substantial age range (18–65 years), aiming to identify whether age, sex, or background experience in sports, music, or video games were associated with aspects of imagery in the visual, auditory, or motor stimulus modality and voluntary or involuntary intentionality level. The findings show weak positive associations between age and increased vividness of voluntary auditory imagery and decreased involuntary musical imagery frequency, weak associations between being female and more vivid visual imagery, and relations of greater music and video game experience with higher involuntary musical imagery frequency. Moreover, all imagery stimulus modalities were associated with each other, for both intentionality levels, except involuntary musical imagery frequency, which was only related to higher voluntary auditory imagery vividness. These results replicate previous research but also contribute new insights, showing that individual differences in age, sex, and background experience are associated with various aspects of imagery such as modality, intentionality, vividness, and frequency. The study’s findings can inform the growing domain of applications of mental imagery to clinical and pedagogical settings.

**Supplementary Information:**

The online version contains supplementary material available at 10.3758/s13421-021-01209-7.

## Introduction

Mental imagery supports several aspects of healthy as well as pathological cognition and has received considerable interest in cognitive psychology research. Different kinds of imagery relate to a range of processes such as memory recall and future thinking (Moulton & Kosslyn, 2009; Schacter et al., 2007), decision-making (Pham et al., 2001), navigation (Bocchi et al., 2017), and mental training (Clark et al., 2012), but also pathological symptomatology (e.g., obsessive-compulsive disorder, posttraumatic stress disorder; American Psychiatric Association, 2013). Pearson (2007) describes mental imagery as the simulation or re-creation of perceptual experience in the absence of a corresponding direct external stimulus from the physical environment. Similar to perception and action, imagery can be experienced in different sensory and stimulus modalities, for example, the visual, auditory (music, speech, and environmental/artificial sounds), olfactory, gustatory, and tactile, as well as movement (which is thought to include proprioceptive and visual elements). Additionally, imagery onset can be voluntary – when we deliberately generate a specific image, as well as involuntary – when imagery emerges in the mind spontaneously, with no intention to experience it. Imagery imitates perception (or action) in several ways. Although there are certain differences between the two in underlying cognitive mechanisms and neural brain areas, the similarity between perception or action and imagery is evident in the overlap of the brain areas that are active for visual (e.g., Chen et al., 1998; Ishai et al., 2000; Johnson & Johnson, 2014; Kosslyn et al., 1999; O'Craven & Kanwisher, 2000; Stokes et al., 2009), auditory (e.g., Aleman et al., 2005; Halpern, 2001; Herholz et al., 2012; Schaefer et al., 2011, 2013; Tian et al., 2018; Watanabe et al., 2020), and motor modalities (Jeannerod, 2001; Munzert et al., 2009).

Despite substantial progress in research on various aspects of imagery, such as the typology of stimulus modality and intentionality level (cf. Schaefer, 2014b, 2017), possible functions (Schacter et al., 2007), and neural correlates (Kosslyn et al., 1999), there are still important questions to be answered. How do specific aspects of imagery such as vividness, or frequency, in different stimulus modalities and intentionality levels, relate to individual characteristics such as age, sex, and background experience in an activity? To what extent do different stimulus modalities and intentionality levels of imagery relate to each other? These questions are of particular importance for obtaining a more cohesive account of the imagery experience, rather than focusing only on a unimodal perspective. Although previous work has looked at individual differences in, for example, musical imagery (Bailes, 2007, 2015; Beaty et al., 2013), investigating imagery in different stimulus modalities and intentionality levels in a single study would have important implications for applied research to further harness imagery’s full potential, for example, in clinical settings (e.g., as part of Cognitive Behavioural Therapy; Pearson et al., 2013), and in pedagogy for mental training and skill acquisition (Halpern & Overy, 2019). The current study attempted to shed light on these issues.

### Aspects of mental imagery

Imagery is conceptualized and studied as a multidimensional experience that incorporates various aspects. We here refer to these aspects of imagery as any characteristic of an imagery type, such as stimulus modality, intentionality, phenomenological qualities such as vividness, or descriptives such as frequency of occurrence or use. The degree to which the literature addresses different aspects of imagery varies depending on the level of intentionality of the imagery. For example, studies on voluntary imagery most commonly measure its vividness. The aspect of vividness refers to the clarity and realism of the imagery (Childers et al., 1985), or its similarity to the actual percept or movement (Lacey & Lawson, 2013; Marks, 1973). In contrast, studies on involuntary imagery have primarily focused and measured aspects directly related to the everyday experience such as its frequency – that is, how often it occurs over a period of time (Floridou et al., 2015; Ortiz de Gortari & Griffiths, 2016). However, which individual factors relate to these aspects of imagery is still unclear.

### Individual differences in mental imagery

Individuals use imagery to varying degrees in everyday life. From visualizing simple daily tasks such as a shopping list (Bassett et al., 2008) to more complicated activities such as mentally rehearsing before a concert the musical material and the movements associated when playing the instrument (Bailes, 2007; Clark et al., 2012), imagery is an integral supporting function for these processes. However, it is worth noting that a small percentage of the general population reports that they cannot voluntarily form visual imagery, a condition termed “aphantasia” (Zeman et al., 2015). As such, individual differences in imagery have been mostly studied in relation to general characteristics common to everyone, such as the demographics of age and sex, and more specifically to activities that likely involve imagery use, such as background experience in sports or music.

Regarding demographics, and more specifically age, the evidence is currently unclear on whether aspects of voluntary imagery (e.g., vividness) or involuntary imagery (e.g., frequency) are part of the processes that deteriorate with age, such as working memory, or of processes that generally do not decline, such as vocabulary or world knowledge (cf. Park et al., 2002). The few existing findings on voluntary auditory and motor imagery suggest that reported vividness is not related to age (Lima et al., 2015; Malouin et al., 2010; Willander & Baraldi, 2010). Involuntary musical imagery frequency appears to decrease as age increases (Floridou et al., 2019; Liikkanen, 2012), although Bailes (2015) reported an increase; however, this study investigated involuntary and voluntary musical imagery conjointly. To our knowledge there are no corresponding studies regarding everyday involuntary visual imagery. These results suggest that specific aspects of imagery in different imagery stimulus modalities may be differentially associated with aging.

Findings related to how the sexes experience imagery are mixed. Some studies show that females report higher imagery vividness than males for the voluntary visual (Campos & Fuentes, 2016; Halpern, 2015; McKelvie, 1995) and auditory (Sacco & Reda, 1998) modalities, which could indicate either differences in processes or in reporting styles between the sexes. However, other studies find no differences between the sexes for voluntary visual and motor imagery, and voluntary as well as involuntary auditory imagery (Campos & Campos-Juanatey, 2014; Campos & Fuentes, 2016; Campos & Pérez-Fabello, 2011; Ernest, 1983; Floridou et al., 2015; Gissurarson, 1992; Halpern, 2015; Sheehan, 1967b; Willander & Baraldi, 2010).

Imagery is an important cognitive tool for mental training in various activities; therefore, its link to background experience in a related domain has received a fair amount of research attention. Individual differences in imagery and background experience in sports and music have been studied extensively. Findings from sports research show that athletes report more vivid visual and motor imagery than do non-athletes (Di Corrado et al., 2014; Hall, 2001; Isaac & Marks, 1994), whilst musicians report more vivid auditory imagery than non-musicians (Aleman et al., 2000; Campos & Fuentes, 2016; Cohen et al., 2011; Hishitani, 2009; Hubbard, 2010; Janata & Paroo, 2006; Keller & Koch, 2008; Moreno et al., 2008; Oxenham et al., 2003; Seashore, 1938). Individuals with increased musical training report more frequent involuntary musical imagery (Hyman Jr. et al., 2013), although engagement with music (e.g., going to gigs, listening to music), not necessarily associated with formal musical training, is a stronger predictor of the occurrence of this type of imagery (Liikkanen, 2012). An additional promising population for research on background experience and imagery are individuals who play video games. Gamers make extensive use of voluntary visual and motor imagery when playing and in preparation for video games (Achtman et al., 2008), and report frequent involuntary visual and musical imagery related to the video game in their everyday life (Game Transfer Phenomena; Ortiz de Gortari, 2019). However, this population has been largely neglected so far in the imagery literature.

Research into individual differences in background experience and imagery suffers from important issues that restrict the generalizability of the findings. First, the samples studied are mostly students, meaning that the range of ages and years of experience in an activity are limited. Secondly, the measurement of the variable of background experience across studies is characterized by several weaknesses: (a) it is primarily investigated as a binary factor, for example, comparison of athletes versus non-athletes or musicians versus non-musicians; (b) the criteria used to define comparison groups vary greatly between studies (i.e., the number of training years); and (c) the studies do not take into consideration individuals who might not be professionals or students but engage with the activity informally as part of a hobby. Our study took a novel approach and explored a sample with a wide age-range and used a background experience index that reflected the frequency, duration, and recency of engagement in various activities such as sports, music, and video games. Using this approach, we aimed to investigate how certain demographics and general background experience in an activity are linked to imagery aspects.

### Imagery modalities and intentionality levels

Research on imagery to date is typically confined to a single stimulus modality (e.g., specific perceptual modalities or the motor domain), mostly the visual, and a single intentionality level, usually the voluntary. However, imagery can be conceptualized as unimodal or multimodal, when it includes, respectively, single or multiple stimulus modalities concurrently or in succession (e.g., when visual imagery of someone playing a musical instrument overlaps with the auditory imagery of the music the instrument produces or followed by the clapping of the audience). Multimodal imagery is often identified as most useful in practical settings such as pedagogy (e.g., Davidson-Kelly et al., 2015; Nanay, 2018). Existing studies and questionnaires that acknowledge and partly address the multiple unimodal imagery types are the Bett’s Questionnaire upon Mental Imagery (QMI; Betts, 1909), a shorter version of QMI (Sheehan, 1967a), and the Plymouth Sensory Imagery Questionnaire (Andrade et al., 2014), all of which measure vividness of imagery in several modalities. These studies and questionnaires provide support both for modality-general and modality-specific imagery mechanisms. Initial findings from studies that have used self-report measures other than the above suggest that visual, auditory, motor, and spatial imagery aspects such as vividness are all associated with each other (Tarampi et al., 2015; for overviews, see Hubbard, 2013, 2019; Hubbard & Ruppel, 2021).

The link between visual and auditory imagery is demonstrated in self-report studies (Campos, 2017; Campos & Pérez-Fabello, 2011; Gissurarson, 1992; Willander & Baraldi, 2010) as well as in neuroimaging findings where activity in specific brain networks (Daselaar et al., 2010; McNorgan, 2012) and individual areas (Lima et al., 2015) underlie both stimulus modalities. Nevertheless, neural areas activated differentially for specific modalities have also been reported (Daselaar et al., 2010; McNorgan, 2012), in many cases overlapping with modality-specific areas involved in actual perception or movement (cf. Kosslyn et al., 2001). In addition, Godøy (2019) suggested a possible link between auditory and motor imagery, a suggestion supported by the activation of various neural areas also involved in movement, when musical imagery is experienced (e.g., Zatorre & Halpern, 2005). An explanation that had been previously put forward for the similarities in musical and motor imagery relates to their common temporal aspect (Schaefer, 2014a) as music unfolds in time (Margulis, 2013), and the sharing by temporal and movement processing of multiple brain areas (cf. Schubotz et al., 2000; Teki et al., 2011).

A one-sided approach in research, similar to focusing on only a single imagery modality, is also seen with regard to the intentionality of imagery initiated voluntarily rather than involuntarily; there is a substantially larger body of work investigating deliberate, effortful imagery as compared to work focusing on everyday and common cases of spontaneous, involuntary imagery. Up until recently, researchers did not distinguish between involuntary and voluntary experiences, generally grouping both experiences together. Therefore, similarities between imagery modalities were attributed to the modality of the perceived stimulus and not the level of intentionality of the imagery. Furthermore, involuntary imagery was mostly studied in subsamples of the population as part of psychopathology symptoms typically related to intrusive thoughts and memories (Berry & Laskey, 2012; Holmes et al., 2004; Smith, 2018), hallucinations in conditions such as in Parkinson’s disease, post-traumatic stress disorder, and schizophrenia (Benson & Park, 2013; Bryant & Harvey, 1996; Matthews et al., 2014; Shine et al., 2015), as well as in conditions such as Charles Bonnet Syndrome and aura in migraines (Jan & del Castillo, 2012; Schott, 2007), and to atypical conditions such as synesthesia (Craver-Lemley & Reeves, 2013). One of the few everyday common, non-clinical forms of involuntary imagery is musical imagery that comes to mind spontaneously and repeatedly. Also known as “an earworm” (Beaman & Williams, 2010; Williamson et al., 2012), this form of imagery has been studied extensively and in relation to other involuntary and voluntary mental processes. The few available findings suggest similarities between the frequency of involuntary musical imagery and other forms of involuntary and voluntary cognition, such as spontaneous mind-wandering (Floridou, 2016), pointing out to a potential intentionality-general mechanism (e.g., retrieval-specific mechanism) underlying voluntary and involuntary cognition forms. Furthermore, relations between the frequency of involuntary musical imagery and the vividness of voluntary auditory imagery have also been reported (Floridou et al., 2015), which could be attributed to the same stimulus modality. This underlines that further research is needed on everyday common forms of mental imagery.

Taken together, the findings suggest that there may be both general and specific imagery mechanisms and processes, related to the stimulus modality and intentionality level of imagery. However, which aspects of imagery are most closely associated with each other is still unclear as there are no studies investigating a range of imagery in various combinations of stimulus modalities and intentionality levels. Most research on imagery has been confined within the boundaries of a single modality and intentionality level. In this study, we included measures of imagery in various stimulus modalities and intentionality levels rather than studying them in isolation and independently of each other, as has generally been the case in previous research. Our approach has the advantage of increasing conceptual and methodological understanding of imagery experiences.

### The present study

The key goal of the current study was to investigate imagery in three stimulus modalities, the *visual, auditory, and motor,* and two levels of intentionality, *voluntary and involuntary*. The first question that was addressed is whether individual differences in age, sex, and background experience in sports, music, and video games are associated with self-reported imagery aspects, such as vividness and frequency. We anticipated that vividness of voluntary auditory imagery and vividness of voluntary motor imagery would not correlate with age (Lima et al., 2015; Malouin et al., 2010; Willander & Baraldi, 2010), while involuntary musical imagery frequency would decrease with increasing age (Floridou et al., 2019; Liikkanen, 2012). We had no specific predictions regarding the relations between age and involuntary or voluntary visual imagery due to the absence of relevant literature. Regarding sex, we predicted that females would report increased vividness of visual imagery compared to men (Campos & Fuentes, 2016; Halpern, 2015; McKelvie, 1995), whilst for auditory and motor imagery we predicted no association with sex (Campos & Campos-Juanatey, 2014; Campos & Fuentes, 2016; Ernest, 1983; Gissurarson, 1992; Halpern, 2015; Sheehan, 1967b; Willander & Baraldi, 2010).

In line with previous findings, we expected that increased background experience with sports would associate with higher vividness of visual and motor imagery (Di Corrado et al., 2014; Hall, 2001; Isaac & Marks, 1994), increased background experience with music would correlate with higher vividness of voluntary auditory imagery and frequency of involuntary musical imagery (Aleman et al., 2000; Campos & Fuentes, 2016; Cohen et al., 2011; Hishitani, 2009; Hubbard, 2010; Janata & Paroo, 2006; Keller & Koch, 2008; Liikkanen, 2012; Moreno et al., 2008; Oxenham et al., 2003; Seashore, 1938), and background experience with video games, which are usually accompanied with musical soundtracks, would positively associate with increased involuntary visual and musical imagery frequency (Ortiz de Gortari, 2019). In addition, we expected that domain-specific background experience would account for any relation observed between the relevant imagery stimulus modality and age and sex, as findings show that engagement with an activity decreases with age (e.g., older ages report lower levels of musical sophistication, which is a multi-faceted concept encompassing several music-related skills and behaviors, including formal musical training, as well as engagement with music at an informal level; Müllensiefen et al., 2014) and for some activities, there are sex biases (e.g., males report increased video game engagement; Shaw, 2012)

The second question we examined is whether there is a relation between imagery stimulus modalities and intentionality levels. Based on insights from previous studies, we hypothesized that there would be positive correlations between the vividness of visual and auditory imagery (Campos, 2017; Campos & Pérez-Fabello, 2011; Gissurarson, 1992; Tarampi et al., 2015; Willander & Baraldi, 2010), as well as between the vividness of auditory vividness and motor imagery (Schaefer, 2014a; Tarampi et al., 2015). In terms of intentionality, we predicted that voluntary auditory imagery vividness would correlate to frequency of involuntary musical imagery (Floridou, 2016; Floridou et al., 2015), while we had no prediction concerning the relation across other intentionality levels due to the lack of literature.

To answer the above questions, we employed an online self-report battery of psychometrically valid and reliable questionnaires about imagery in the visual, auditory, and motor stimulus modalities, in both intentionality levels where possible. Moreover, we assessed information about age, sex, and background experience in sports, music, and video games in a sample representative of the Dutch population in terms of age and sex.

## Method

### Participants

Participants were recruited via social media, recruitment websites, personal networks, and posters at various public spaces (e.g., university library) in the Netherlands. A total of 690 individuals commenced participation in the online survey and of these 296^1^ completed all questions. The questionnaires related to imagery were part of a larger survey also examining placebo- and nocebo-like effects, which were not analyzed for the purposes of the present study. We excluded 17 participants (i.e., 5.7% of the data) based on quality criteria either related to the wider survey or specific to the imagery study, which were the following: (a) total survey completion duration (less than one-third of the median duration of 33 min; five participants); (b) an indication of not reading and/or not understanding all or most of the questions (based on two items at the end of the survey; one participant); (c) incorrect responses to one or two (out of two) attention filter questions (nine participants); (d) non-fluency in Dutch (based on an item in the demographic section of the survey; one participant); and (e) missing age (one participant). The total sample included in the final analysis consisted of 279 participants (149 females, 53.41%) ranging in age from 18 to 65 years (*M =* 41.12, *SD =* 14.18), and was representative of the Dutch population in terms of age and sex.^2^ The completion rate was similar to that of other online studies (Burgard et al., 2020; Floridou et al., 2019; Müllensiefen et al., 2014; Peerdeman et al., 2018). The sample was primarily of Dutch nationality (94.27% Dutch, 2.15% multiple, 3.58% other) and generally highly educated (65.95% tertiary, 33.69% secondary, 0.36% primary). Participants who completed all questions could opt to take part in a gift voucher raffle (1 × €100, 10 × €20).

### Ethics statement

The larger study received ethical approval from the Psychology Research Ethics Committee of Leiden University, the Netherlands (application number CEP 16-0226/99). All participants provided informed consent online via checkboxes on the first page of the survey before commencement of the study.

### Materials

#### Measures

We administered the following battery of self-report imagery questionnaires in the Dutch language^3^:

##### Visual imagery

The Vividness of Visual Imagery Questionnaire (VVIQ; Marks, 1973) measures the vividness of visual imagery. Participants are asked to imagine four scenes (relative/friend, rising sun, a shop, and a landscape) and visualize four different aspects of each (e.g., color, shape), amounting to 16 items in total. Vividness ratings range from 1 *(Perfectly clear and as vivid as normal vision)* to 5 (*No image at all, you only “know” you are thinking of the object*). The instructions ask participants to imagine the scenes with their eyes closed. In the first validation study (Campos et al., 2002) that followed the development of the original questionnaire (Marks, 1973), good internal consistency was found (*α* = .88).

The Spontaneous Use of Imagery Scale (SUIS; Reisberg et al., 2003) measures the general occurrence of imagery in everyday life. The original scale has 12 items (e.g., “When I think about visiting a relative, I almost always have a clear mental picture of him or her*”*), which measure a general factor, and uses a 5-point rating scale (1 = *Never appropriate*; 5 = *Always completely appropriate*). In the validation study of the Dutch version (Nelis et al., 2014), items 1, 3, and 6 were excluded based on their low psychometric qualities, leaving a total of nine items with good internal consistency (*α* = .73), which was adopted for the current study as well.

##### Auditory imagery

The Bucknell Auditory Imagery Scale (BAIS; Halpern, 2015) is a self-report measure of voluntary auditory imagery for musical, verbal, and environmental sounds, with two subscales, *Vividness* (BAIS-V) and *Control* (BAIS-C). Each subscale has 14 items,^4^ which prompt participants to construct auditory mental imagery and rate it on Vividness (e.g., “a trumpet playing the opening of ‘Happy Birthday’”) and Control (e.g., “the ease of imaging a change from a trumpet to a violin*”*) on a 7-point scale (*Vividness*: 1 = *No image present at all*; 7 = *As vivid as the actual sound*; *Control*: 1 = *No image present at all*; 7 = *Extremely easy to change the image*). The original validation study (Halpern, 2015) reports good internal consistency for both subscales (BAIS-V, *α* = .83; BAIS-C, *α* = .81). In the current study we only included the Vividness subscale in the analysis, given the focus of the other utilized measures.

The Involuntary Musical Imagery Scale (IMIS; Floridou et al., 2015) measures four phenomenological characteristics of recurring involuntary musical imagery (Negative Valence, Movement, Personal Reflections, and Help*;* 15 items; 5-point response scale from 1 = *Never* to 5 = *Always*). In addition, three items independent of the scale but commonly used alongside it measure other characteristics of involuntary musical imagery such as frequency of retrieval (1 = *Never* to 6 = *Almost continuously*), duration of the section (e.g., chorus, verse, entire piece) of the piece of music retrieved (1 = *Less than 5 seconds* to 5 = *More than 1 minute*), and duration of the episode (i.e., a period of time when one particular musical section and any additional sections of the same piece appears and is repeated; 1 = *Less than 10 minutes* to 5 = *More than 2 hours*). Only involuntary musical imagery frequency was used for the purposes of our study.

##### Motor imagery

The Vividness of Movement Imagery Questionnaire (VMIQ-2; Roberts et al., 2008) assesses vividness of movement in three different ways: (1) third-person imagery perspective of the self, as if the individual is watching themselves performing the movement (*External Visual Imagery*, VMIQ-EVI); (2) first-person visual imagery perspective, as if the individual is looking out through their own eyes while performing the movement (*Internal Visual Imagery*, VMIQ-IVI); and (3) the feeling of carrying out the movement (*Kinesthetic Visual Imagery*, VMIQ-KVI). VMIQ-2 has 12 items and their response ratings are on a 5-point scale (1 = *Perfectly clear and vivid as normal vision/feel of movement* to 5 = *No image at all, you only know that you are thinking of the skill*). The instructions ask participants to imagine and rate the vividness of items first in VMIQ-EVI, then VMIQ-IVI, and finally VMIQ-KVI. In the original validation study (Roberts et al., 2008), high internal consistency was found for all subscales (VMIQ-EVI, *α* = .95; VMIQ-IVI, *α* = .95; VMIQ-KVI, *α* = .93).

##### Background experience

Background experience in sports (sports in general, including dancing), music (playing an instrument/singing), and video games (video games of any kind) was measured with three items for each activity: (1) frequency of engaging in the activity during the last year (response scale from 1 = *Never* to 5 = *Daily*); (2) duration (in months and years) of engaging in the activity at least once a week (open-ended); (3) recency (in months and years) of engaging in the activity at least once a week (open-ended).

### Procedure

The overall survey was implemented online in Qualtrics (https://www.qualtrics.com). First, participants read the information about the study and provided their consent to participate. The presentation of the consent form and demographic questions was in a fixed order, whilst the presentation of the imagery questionnaires was randomized. At the end, participants were debriefed about the purposes of the studies. The median total completion duration of the survey was 33 min (including the measures taken for the larger study mentioned above).

### Statistical analyses

First, we reversed the scores of all items of VVIQ and VMIQ-2 as indicated by the scoring system of each questionnaire. Next, we calculated the scores of each measure and its subscales based on the sum of its items. Then, we developed a background experience index for each domain separately. We did this by calculating the sum of three items related to background experience, that is, frequency, duration (values converted to months and then normalized by dividing each score with the maximum value of the participant pool), and recency (values converted to months, then reversed as lower numbers indicated more recent involvement and then normalized by dividing each value by the maximum score of the participant pool). In the Online Supplementary Material (Tables 1, 2 and 3) we also report descriptives and further analyses with the composite scores we calculated for the following variables: BAIS Total (based on the average scores of the two subscales BAIS-V and BAIS-C) and VMIQ-2 Total (by adding up the scores of the three subscales of VMIQ-2 EVI, IVI, KVI).

We analyzed the data using IBM SPSS Statistics (Version 24). We ran Spearman correlations to investigate associations of age, sex, and background experience with imagery aspects such as vividness and frequency, as the data were not normally distributed. We corrected for multiple testing using the Holm-Sidak method (Aickin & Gensler, 1996). Using the *r* metric, effect sizes of .10, .30, and .50 were considered small, medium, and large, respectively (Cohen, 1988). We ran partial correlations to explore if any relations observed between age or sex and imagery were explained by background experience in the relevant imagery modality.

## Results

### Descriptive statistics

**Fig. 1:**
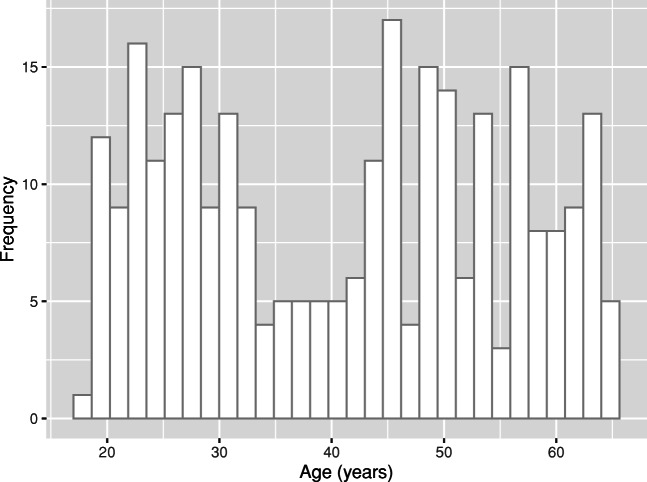
Histogram displaying the distribution of participants’ ages (Skewness *SE* = .15, Kurtosis *SE* = .29)

Descriptive statistics are presented to provide context and opportunities for comparisons to previous literature. The presentation of all descriptives from the imagery measures is in Table 1. The current descriptives and Cronbach’s alpha reliability values for all imagery measures are comparable to those found in the original measurement validation studies as reported in the *Materials* section. In our sample, 2.5% of participants scored ≤ 30 in VVIQ, which is the threshold score for individuals with aphantasia (Wicken et al., 2019; Zeman et al., 2015), in line with previous reports of its prevalence, estimated to lie around 2.4% of the general population (Faw, 2009). In addition, in Fig. 1 we present a histogram displaying the distribution of participants’ ages. There was a relatively large age range in our sample (18–65 years), and a bimodal distribution with a satisfactory representation of all age groups. Finally, the presentation of descriptive statistics for the background experience indices is given in Table 2 (the sample sizes for each background experience index vary due to the exclusion of participants with no background experience in each activity).

**Table 1:** Descriptive statistics for all imagery measures (*n* = 279)

Modality	Intentionality	Measure	Aspect	Minimum	Maximum	*M*	*SD*	α
Visual	Voluntary	VVIQ	Vividness	16	80	62.47	10.92	.93
Visual	Involuntary	SUIS	Frequency	11	44	29.57	6.16	.76
Auditory	Voluntary	BAIS-V	Vividness	1.21	7	4.71	1.13	.91
Auditory: Musical	Involuntary	IMIS	Frequency	1	6	3.70	1.36	N/A
Motor: External	Voluntary	VMIQ-EVI	Vividness	12	60	42.86	12.30	.97
Motor: Internal	Voluntary	VMIQ-IVI	Vividness	12	60	46.97	12.30	.96
Motor: Kinesthetic	Voluntary	VMIQ-KVI	Vividness	12	60	46.70	10.80	.96

*Note*. Minimum, Maximum, Mean (*M*), Standard Deviation (*SD*), and Cronbach’s Alpha (*α*)

*VVIQ* Vividness of Visual Imagery Questionnaire, *SUIS* Spontaneous Use of Imagery Scale, *BAIS-V* Bucknell Auditory Imagery Scale - Vividness, *IMIS* Involuntary Musical Imagery Scale (frequency), *VMIQ-EVI* Vividness of Motor Imagery Questionnaire – External Visual Imagery, *VMIQ-IVI* Vividness of Motor Imagery Questionnaire - Internal Visual Imagery, *VMIQ-KVI* Vividness of Motor Imagery Questionnaire - Kinesthetic Visual Imagery

**Table 2:** Descriptive statistics for background experience indices

Background experience indices	Minimum	Maximum	*M*	*SD*
Sports (*n* = 214)	1.81	7.00	5.37	1.19
Music (*n* = 133)	1.03	7.00	4.34	1.82
Video games (*n* = 107)	1.18	6.61	4.18	1.74

*Note.* Minimum, Maximum, Mean (*M),* and Standard Deviation *(SD)*

### Individual differences in imagery: Bivariate correlations

To explore our first research question, concerning the relation of imagery aspects with individual differences, we examined the associations between age, sex, and background experience in sports, music, and video games with all imagery measures (Table 3). Increasing age was weakly associated with higher vividness of voluntary auditory imagery (BAIS-V, *r*(279) = .14, *p* = .017) and with decreased frequency of involuntary musical imagery (IMIS (frequency), *r*(279) = -.12, *p* = .042). Age was not associated with any of the measures related to voluntary and involuntary visual imagery (VVIQ, *r*(279) = .11, *p* = .058; SUIS, *r*(279) = .04, *p* = .507) or voluntary motor imagery (VMIQ-EVI, *r*(279) = .02, *p* = .726, VMIQ-IVI, *r*(279) = -.09, *p* = .12, VMIQ-KVI, *r*(279) = .02, *p* = .668).

**Table 3:** Spearman correlations of all imagery measures with age, sex, and background experience index of sports, music, and video games

Stimulus	Intentionality	Measure	Age (*n* = 279)	Sex (female; *n*=279)	Background experience index		
					Sports (*n* = 214)	Music (*n* = 133)	Video games (*n* = 107)
Visual	Voluntary	VVIQ	.11	.15*	.07	.11	.03
Visual	Involuntary	SUIS	.04	.19**	-.01	.11	.03
Auditory	Voluntary	BAIS-V	.15*	-.05	.04	.15	-.03
Auditory: Musical	Involuntary	IMIS	-.12*	-.03	.09	.30**	.25**
Motor	Voluntary	VMIQ-EVI	.05	-.01	.10	.01	-.06
		VMIQ-IVI	-.06	-.05	.04	.03	.10
		VMIQ-KVI	.05	.05	.08	-.004	.12

*VVIQ* Vividness of Visual Imagery Questionnaire, *SUIS* Spontaneous Use of Imagery Scale, *BAIS-V* Bucknell Auditory Imagery Scale - Vividness, *IMIS* Involuntary Musical Imagery Scale (frequency), *VMIQ-EVI* Vividness of Motor Imagery Questionnaire – External Visual Imagery, *VMIQ-IVI* Vividness of Motor Imagery Questionnaire - Internal Visual Imagery, *VMIQ-KVI* Vividness of Motor Imagery Questionnaire - Kinesthetic Visual Imagery

* p < .05. ** *p* < .01

We observed weak correlations between sex and voluntary as well as involuntary visual imagery (VVIQ, *r*(279) = .15, *p* = .010; SUIS, *r*(279) = .20, *p* = .001), indicating that females reported experiencing more vivid voluntary and more frequent involuntary visual imagery. We found no relations between sex and vividness of voluntary auditory and motor imagery nor frequency of involuntary musical imagery (BAIS-V, *r*(279) = -.03, *p* = .568; VMIQ-EVI, *r*(279) = -.016, *p* = .795; VMIQ-IVI, *r*(279) = -.06, *p* = .355; VMIQ-KVI, *r*(279) = .04, *p* = .486; IMIS (frequency), *r*(279) = -.03, *p* = .599).

The background experience indices regarding music and video games were moderately and weakly associated with increased frequency of involuntary musical imagery (IMIS (frequency), *r*(132) = .30, *p* < .001 and *r*(104) = .25, *p* = .010, respectively). We did not observe any further relations.

In Tables 1 and 2 of the Online Supplementary Material we present descriptives of and correlations between all subscales of the imagery measures we used and which are not presented here (e.g., BAIS-C), as well as composite scores of scales (e.g., BAIS Total, VMIQ-2 Total), with age, sex, and background experience in sports, music, and video games.

### Individual differences in imagery: Partial correlations

To follow up our first research question we explored whether background experience in an activity could explain the relations of age and sex with imagery aspects. First, we calculated partial correlations between age and vividness of voluntary auditory imagery (BAIS-V), as well as frequency of involuntary musical imagery (IMIS frequency), while controlling for musical experience (Background Experience Index). When controlling for background experience in music, the magnitude of the correlation between age and vividness of voluntary auditory imagery (BAIS-V, *r*(130) = .15, *p* = .090), and age and frequency of involuntary musical imagery (IMIS (frequency), *r*(130) = -.13, *p* = .150), remained approximately unchanged, indicating that musical experience does not explain the relations between age and vividness of voluntary auditory imagery nor frequency of involuntary musical imagery. Next, we explored whether the background experience in video games could partially account for the relation of sex with vividness of visual imagery. The magnitude of the correlations remained approximately unchanged, indicating that background experience did not account for the relation of sex with vividness of voluntary (VVIQ; *r*(104) = .17, *p* = .089) nor frequency of involuntary visual imagery (SUIS; *r*(104) = .22, *p =* .023).

### The relation between imagery stimulus modalities and intentionality levels: Bivariate correlations

To explore our second research question, concerning the relations between imagery modalities and intentionality levels, we calculated correlations between all imagery measures (Table 4). First, we see that, within stimulus modalities but across intentionality levels, the visual imagery measures correlated moderately with each other (VVIQ and SUIS, *r*(279) = .31, *p* < .001) and the auditory measures correlated weakly with each other (BAIS-V and INMI frequency, *r*(279) = .14, *p* = .021). Concerning stimulus modalities, we observed primarily strong and some moderate correlations between all modalities, that is, visual and auditory imagery (VVIQ and BAIS-V: *r*(279) = .46, *p* < .001; SUIS and BAIS-V*: r*(279) = .32, *p* < .001), visual and motor imagery (VVIQ and VMIQ-EVI: *r*(279) = .43, *p* < .001; VVIQ and VMIQ-IVI: *r*(279) = .41, *p* < .001; VVIQ and VMIQ-KVI: *r*(279) = .48, *p* < .001; SUIS and VMIQ-EVI: *r*(279) = .25, *p* < .001; SUIS and VMIQ-IVI: *r*(279) = .25, *p* < .001; SUIS and VMIQ-KVI: *r*(279) = .20, *p* = .001) as well as auditory and motor imagery (BAIS-V and VMIQ-EVI: *r*(279) = .46 , *p* < .001); BAIS-V and VMIQ-IVI: *r*(279) = .50 , *p* < .001); BAIS-V and VMIQ-KVI: *r*(279) = .46 , *p* < .001).

**Table 4:** Spearman correlations between all mental imagery measures (*n* = 279)

	VVIQ	VMIQ-EVI	VMIQ-IVI	VMIQ-KVI	BAIS-V	IMIS
SUIS	.31**	.25**	.25**	.20**	.32**	.10
VVIQ		.43**	.41**	.48**	.46**	.00
VMIQ-EVI			.67**	.50**	.46**	.06
VMIQ-IVI				.68**	.50**	.14*
VMIQ-KVI					.46**	.11
BAIS-V						.14*

*SUIS* Spontaneous Use of Imagery Scale, *VVIQ* Vividness of Visual Imagery Questionnaire, *VMIQ-EVI* Vividness of Motor Imagery Questionnaire – External Visual Imagery, *VMIQ-IVI* Vividness of Motor Imagery Questionnaire - Internal Visual Imagery, *VMIQ-KVI* Vividness of Motor Imagery Questionnaire - Kinesthetic Visual Imagery, *BAIS-V* Bucknell Auditory Imagery Scale - Vividness, *IMIS* Involuntary Musical Imagery Scale (frequency)

* *p* < .05*.* ** *p* < .01

In relation to intentionality, we see that all voluntary measures correlated moderately to strongly with each other (VVIQ and BAIS-V: *r*(279) = .46, *p* < .001; VVIQ and VMIQ-EVI: *r*(279) = .43, *p* < .001; VVIQ and VMIQ-IVI: *r*(279) = .41, *p* < .001; VVIQ and VMIQ-KVI: *r*(279) = .48, *p* < .001; BAIS-V and VMIQ-EVI: *r*(279) = .46 , *p* < .001; BAIS-V and VMIQ-IVI: *r*(279) = .50 , *p* < .001; BAIS-V and VMIQ-KVI: *r*(279) = .46 , *p* < .001). The involuntary measures (SUIS and IMIS frequency: *r*(279) = .10, *p* = .11) did not correlate with each other. With regard to relations between intentionality measures, we see that involuntary visual imagery (SUIS) correlated weakly to moderately with all voluntary measures (visual imagery, VVIQ: *r*(279) = .31, *p* < .001; auditory imagery, BAIS-V: *r*(279) = .46, *p* < .001; motor imagery, VMIQ-EVI: *r*(279) = .25, *p* < .001; VMIQ-IVI: *r*(279) = .25, *p* < .001; VMIQ-KVI: *r*(279) = .20, *p* < .001), whilst, as reported above, involuntary musical imagery (IMIS frequency) correlated weakly only with voluntary auditory imagery (BAIS-V and IMIS (frequency), *r*(279) = .14, *p* = .021).

## Discussion

The main question of the current study was whether individual differences in age, sex, and background experience in sports, music, and video games are associated with self-reported aspects of imagery in various modality and intentionality levels. First, we found that increasing age was weakly associated with higher vividness of voluntary auditory imagery and lower frequency of involuntary musical imagery, but not with any of the other imagery stimulus modalities and intentionality levels. Second, females reported more vivid voluntary and more frequent involuntary visual imagery. Third, more background experience with music as well as video games were associated with increased frequency of involuntary musical imagery, but no other relations were seen for background experience and imagery. Finally, we found that background experience in a specific activity did not account for any of the observed relations of the demographics of age and sex with various imagery aspects.

The second question of our study was whether there is a relation between imagery stimulus modalities and intentionality levels. All stimulus modalities (visual, auditory, and motor) did correlate with each other, as well as with all intentionality levels (voluntary and involuntary) within modalities, except for involuntary musical imagery frequency, which only correlated with vividness of voluntary auditory imagery. These findings replicate previous results but also bring some novel key information. Below we synthesize the findings in relation to the existing literature and discuss their implications for future research.

### Individual differences in imagery

The slight increase of reported vividness of voluntary auditory imagery with age that we observed is puzzling as previous research (Lima et al., 2015) using the same measure did not find such a relation, while corresponding correlations for imagery with age in other stimulus modalities did not follow the same direction. A research project with two studies on the topic (Schenker, 2018) found support for both our and Lima et al.’s (2015) findings. One potential explanation could lie in the difference between the sample size of our study (*n* = 279) and the Lima et al. (2015) study (*n* = 74), with the latter being too small to detect weaker effects of individual differences. Other possible explanations could relate to differences in other demographic factors between the studies such as age range, education level, and culture, which future studies should investigate. Our findings should also be interpreted with caution, as the size of the correlation is small and of course it does not imply causality. Interestingly, age was not associated with vividness of visual imagery. As previous research has reported age-related reductions in other visual imagery aspects such as manipulation (Craik & Dirkx, 1992), and rotation and maintenance (Dror & Kosslyn, 1994), this could further indicate that different imagery aspects or levels of abstraction in the task (i.e., naturalistic voluntary imagery tasks vs. abstract experimental imagery tasks) within the same modality depend on separate mechanisms differentially associated with aging. Clearly, further research is needed to assess various imagery aspects in relation to aging. Increasing age was weakly associated with reductions in frequency of involuntary musical imagery, a finding that corresponded with our expectations and most previous literature (Floridou et al., 2019; Liikkanen, 2012). An increasing number of studies suggest a reduction in the reported frequency of involuntary cognition with age (Maillet & Schacter, 2016; Seli et al., 2017), which has been attributed to a general decrease in cognitive resources in older adults (Craik, 1986). However, this suggestion cannot explain the lack of relation between age and the remaining imagery measures (or even weak increases for voluntary auditory imagery), which could be an indication of how the frequency of involuntary imagery, as opposed to other aspects of imagery, for example vividness, relies on different cognitive systems that may be differentially associated with aging.

Next, we investigated the relation between sex and imagery. Our findings agree with existing literature and confirm our hypotheses that females report higher vividness of voluntary visual imagery (Campos & Fuentes, 2016; Halpern, 2015; McKelvie, 1995) and that there would be no relation with the other stimulus modalities and intentionality levels (Campos & Campos-Juanatey, 2014; Campos & Fuentes, 2016; Campos & Pérez-Fabello, 2011; Ernest, 1983; Gissurarson, 1992; Halpern, 2015; Sheehan, 1967b; Willander & Baraldi, 2010). Our findings also extend the literature by demonstrating that females report more involuntary visual imagery. An explanation for females reporting increased vividness of visual imagery but no other stimulus modalities in either intentionality level could suggest that vividness of visual imagery taps into different mechanisms than the other imagery modalities. This finding could also be attributed to sex hormones, and more specifically progesterone, which has been attributed a role in visual imagery vividness (Wassell et al., 2015) and visual perception (Broverman et al., 1981; Wijayanto et al., 2009).^5^ Finally, a methodological issue worth noting is that, in research on sex, gender, and imagery, the exact questions posed to participants are rarely reported and that, when gender is asked, the findings reported are mostly binary. Furthermore, in many such cases, sex and gender are used interchangeably, which may affect the results with regard to both how participants define themselves, and how the results are interpreted in terms of cognitive differences in relation to sex and gender.

Regarding domain-specific background experience, we observed small to moderate associations only between increased background experience in music and video games, and more frequent involuntary musical imagery. Previous studies have identified similar relations between musical training and engagement, as well as video games use, with higher frequency of involuntary musical imagery (Floridou et al., 2015; Ortiz de Gortari & Griffiths, 2016). A possible speculative explanation for the lack of association between any background experience and other imagery modalities might be that background experience does not necessarily boost self-reported vividness, but does increase the amount of time individuals spend thinking about the relevant activity, even if involuntarily. This relation could only be captured by the item of frequency of involuntary musical imagery, as the remaining measures focus on vividness. Future research should explore this possibility and measure the frequency of imagery in other stimulus modalities in daily life.

Our results about the lack of effect of background experience in music on vividness of auditory imagery agree with Hubbard and Ruppel (2021), who used the same questionnaire as in our study (BAIS), but contradict the findings of previous studies that found increased vividness of non-musical auditory imagery in musicians (Aleman et al., 2000; Campos & Fuentes, 2016; Cohen et al., 2011; Hishitani, 2009; Hubbard, 2010; Janata & Paroo, 2006; Keller & Koch, 2008; Moreno et al., 2008; Oxenham et al., 2003; Seashore, 1938) and visual and motor imagery in athletes (Di Corrado et al., 2014; Hall, 2001; Isaac & Marks, 1994). This discrepancy could be due to the different measures of background experience, as well as measures of imagery, used in previous studies and ours, or to the use of a continuous measure of experience as in our study where we did not find an effect rather than a dichotomous measure (dichotomizing continuous data could inflate type 1 error/false positives to observe a difference that is not apparent in continuous data; Altman & Royston, 2006; Austin & Brunner, 2004) as in previous studies that found an effect. A potential explanation for the lack of associations between imagery aspects and background experience comes from the auditory imagery literature, specifically musical imagery. Interestingly, other studies show low to moderate correlations between musical training and general auditory imagery abilities but higher correlations between musical training and musical imagery (Herholz et al., 2012; Pfordresher & Halpern, 2013; Zatorre et al., 2010). These findings suggest that individuals who are experienced with music score higher in imagery aspects specifically for music (the activity that they have gained experience in), rather than in imagery aspects in the general imagery modality (auditory) or a different one (e.g., visual). Gelding et al. (2015) suggested that it is the use of strategy in musical imagery, rather than simply musical experience, that leads to better performance of musicians in musical imagery tasks. An alternative explanation could be that individuals who do not already experience vivid auditory or musical imagery might not choose music as an activity to acquire experience in, or do not use a musical imagery strategy.^6^ Future studies could explore different types of background experience with an activity (e.g., different types of sports or musical instrument), which could reflect more fine-grained differences between imagery aspects.

Finally, background experience with music and video games did not account for the age-related changes in vividness of voluntary auditory imagery or frequency of involuntary musical imagery, or sex-related changes in voluntary and involuntary visual imagery, respectively. Future studies should explore other factors that have been suggested to explain the relation of imagery with aging and sex, such as meta-awareness of the occurrence and the role of sex hormones, respectively.

### The relation between imagery modalities and intentionality levels

Our second research question concerned the relation between stimulus modalities and intentionality levels of imagery. Our results replicate previous preliminary findings of commonalities between multiple imagery stimulus modalities (cf. Tarampi et al., 2015) and confirm our hypotheses regarding associations between the vividness of visual and auditory as well as auditory and motor imagery modalities. They also extend previous findings demonstrating a strong relation between the vividness of visual and motor imagery. A possible explanation for this could be related to multimodal perception, since our perception of the real world is rarely unimodal (Bertelson & de Gelder, 2004; O’Callaghan, 2014; Spence et al., 2004), suggesting that a similar mechanism may operate in the case of imagery (Nanay, 2018).

Although not directly assessed in our study, mental imagery modalities can frequently co-occur (e.g., visual imagery of a car can be accompanied by auditory imagery of the engine sound; also see Intons-Peterson, 1983; Spence & Deroy, 2013), something that could boost their association even when measured independently of each other as multiple unimodal imagery types. Furthermore, BAIS, which is an auditory imagery measure, in the instructions also uses visual imagery when constructing the context of auditory imagery. As for VMIQ-2, although it measures movement, only one subscale assesses kinesthetic aspects and the rest are associated to visual imagery when observing movement of others or the participant, for example, VMIQ-EVI and VMIQ-IVI, which could also explain the correlations we found between all subscales of VMIQ-2 and VVIQ. Our findings provide support for an underlying stimulus modality-general mechanism in relation to vividness of visual, auditory, and motor stimulus modalities. Previous studies have implicated the long-term memory as well as the working memory as the underlying systems for vividness of visual and auditory imagery (Baddeley & Andrade, 2000). However, more research is needed to extend this hypothesis to motor imagery, preferably measuring imagery in experimental settings (cf. Gelding et al., 2015) at the moment it occurs. If the relations reported here are confirmed and their mechanisms are identified, this would have implications for transfer effects between modalities and relevant health interventions (e.g., in movement rehabilitation where auditory imagery cues are used effectively to regularize movement; Satoh & Kuzuhara, 2008; Schaefer et al., 2014).

Our findings about the level of intentionality and the involvement of general and specific mechanisms are not conclusive. Even though all indices of voluntary imagery were intercorrelated, the two measures of involuntary imagery were not. One potential explanation for this is that the measures we used for visual (SUIS) and auditory musical (IMIS frequency) imagery assess different aspects of intentionality (automatic completion vs. frequency of occurrence, respectively), while voluntary measures focus on the same aspect (e.g., vividness). Future studies should consider and develop robust methods to measure the time course as well as co-occurrence and/or switch between stimulus modalities and intentionality levels. Some imagery occurrences are purely unimodal, or associated with one intentionality level, and others might be multimodal or move on a continuum of intentionality, starting involuntarily and continuing voluntarily or vice versa (for mind-wandering, see Seli et al., 2016; Smallwood, 2013; for musical imagery, see Cotter et al., 2019), as well as for instances that start as visual and switch to auditory imagery.

### Limitations

When discussing the present results, some limitations should be considered. First, the magnitude of the correlations is generally modest and should be interpreted with caution. Second, running multiple correlations, as we did, could increase Type 1 error, although we used the Holm-Sidak method to correct for multiple correlations and interpreted the findings using the r metric instead of alpha values. Third, although we used measures most relevant to the intentionality of imagery it is clear that there is a need for the development of measures of imagery that will take the intentionality aspect into account in relation to all stimulus modalities, as has been done recently in the research of other experiences such as mind-wandering, where studies have used questionnaires distinguishing between the two levels of intentionality and which have revealed different behavioral and neural correlates (Carriere et al., 2013). Although IMIS is straightforwardly about involuntary experiences, SUIS can be considered a mix of involuntary, automatic, as well as the voluntary use of visual imagery, which makes the need for the development of fine-grained measures imperative. Fourth, scores on various imagery stimulus modality scales often correlate quite highly with each other, which could indicate an issue of convergence validity. This could either result from the relation between all imagery stimulus modalities, or be related to the development of the measures representing a considerable overlap in the measure construction, such that they might not be able to distinguish fine-grained differences between stimulus modalities. Finally, an issue inherent in all self-report measures relates to whether the observed relations are truly associated with changes in age, or reflect the reporting style of participants. Future studies should take this into consideration and either provide measures of confidence or social desirability as proxies related to the validity of the reports.

### Conclusions

Our findings demonstrate that individual differences in age, sex, and background experience in a related activity are associated to varying degrees with different aspects of mental imagery. Furthermore, our study supports the idea of stimulus modality general mechanisms, at least for vividness of visual, auditory, and motor modalities; however intentionality-general and -specific mechanisms should be further explored. These findings do not support a need for applications in clinical or pedagogical domains to be adjusted for age (at least within the range included here), and suggest that background experience may in these cases also not give cause to expect large differences in imagery aspects.

## Supplementary Information

ESM 1 (DOCX 96 kb)

## Footnotes

1 For stable correlation estimates n should generally approach 250 (Schönbrodt & Perugini, 2013).

2 Matching the Dutch population age and sex distribution according to the state statistics found here: http://statline.cbs.nl/StatWeb/publication/?VW=T&DM=SLNL&PA=37296ned&D1=a&D2=0,10,20,30,40,50,60,%28l-1%29,l&HD=130605-0924&HDR=G1&STB=T

3 All the measures of which no Dutch translation was yet available were translated into Dutch through a forward- and back-translation procedure (Behling & Law, 2000).

4 In correspondence with the BAIS author (Andrea Halpern), item 6 was changed from a baseball game to a soccer game (more common in the Netherlands) and item 8 was changed from Beethoven’s fifth to “a symphony” (to avoid unfamiliarity). These cross-language and -culture differences are common, as stated by Halpern (2015), who reports similar modifications in other languages.

5 We would like to thank an anonymous reviewer for this suggestion.

6 We would like to thank Tim Hubbard for suggesting this alternative explanation.
