# Supplementary material for: Individual differences in mental imagery in different modalities and levels of intentionality
Source: Mem Cognit. 2021 Aug 30;50(1):29–44. doi: 10.3758/s13421-021-01209-7 (PMC8763825; doi:10.3758/s13421-021-01209-7)
Supplement: Supplementary file 1 — (DOCX 96 kb) [file 13421_2021_1209_MOESM1_ESM.docx]

Table 1

Descriptive statistics for all imagery measures and subscales as well as composite scores *(n = 279)*

| Modality | Intentionality | Measure | Aspect | Minimum | Maximum | *M* | *SD* | *α* |
| --- | --- | --- | --- | --- | --- | --- | --- | --- |
| Visual | Voluntary | VVIQ | Vividness | 16 | 80 | 62.47 | 10.92 | .93 |
| Visual | Involuntary | SUIS | Frequency | 11 | 44 | 29.57 | 6.16 | .76 |
| Auditory | Voluntary | BAIS-TOT | Ability | 1.11 | 7 | 4.90 | 1.07 | .95 |
|  |  | BAIS-V | Vividness | 1.21 | 7 | 4.71 | 1.13 | .91 |
|  |  | BAIS-C | Control | 1 | 7 | 5.08 | 1.13 | .93 |
| Auditory: Musical | Involuntary | IMIS | Frequency | 1 | 6 | 3.70 | 1.36 | N/A |
| Motor | Voluntary | VMIQ-TOT | Vividness | 48 | 180 | 136.53 | 29.12 | .97 |
|  |  | VMIQ-EVI | Vividness | 12 | 60 | 42.86 | 12.30 | .97 |
|  |  | VMIQ-IVI | Vividness | 12 | 60 | 46.97 | 12.30 | .96 |
|  |  | VMIQ-KVI | Vividness | 12 | 60 | 46.70 | 10.80 | .96 |

*Note.* Minimum, Maximum, Mea*n (M),* Standard Deviation *(SD),* and Cronbach’s Alpha (*α*)

*VVIQ* Vividness of Visual Imagery Questionnaire, *SUIS* Spontaneous Use of Imagery Scale, *BAIS-TOT* Bucknell Auditory Imagery Scale - Total, *BAIS-V* Bucknell Auditory Imagery Scale - Vividness, *BAIS-C* Bucknell Auditory Imagery Scale - Control, *IMIS* Involuntary Musical Scale (frequency), *VMIQ-TOT* Vividness of Motor Imagery Questionnaire - Total, *VMIQ-EVI* Vividness of Motor Imagery Questionnaire – External Visual Imagery, *VMIQ-IVI* Vividness of Motor Imagery Questionnaire - Internal Visual Imagery, *VMIQ-KVI* Vividness of Motor Imagery Questionnaire - Kinesthetic Visual Imagery

Table 2

Spearman correlations of all imagery measures with age, sex, and background experience index of sports, music, and video games

|  |  | Measure | Age  (*n* = 279) | Sex  (*n*=279) | Background Experience Index | | |
| --- | --- | --- | --- | --- | --- | --- | --- |
| Modality | Intentionality |  |  |  | Sports  (*n* = 214) | Music  (*n* = 133) | Video games (*n* = 107) |
| Visual | Voluntary | VVIQ | .11 | .15* | .07 | .11 | .03 |
| Visual | Involuntary | SUIS | .04 | .19** | -.01 | .11 | .03 |
| Auditory | Voluntary | BAIS-TOT | .18** | .01 | .10 | .13 | -.01 |
|  |  | BAIS-V | .15* | -.05 | .04 | .15 | -.03 |
|  |  | BAIS-C | .18** | .06 | .13 | .09 | .02 |
| Auditory: Musical | Involuntary | IMIS | -.12* | -.03 | .09 | .30** | .25** |
| Motor | Voluntary | VMIQ-TOT | .01 | -.02 | .07 | .01 | .07 |
|  |  | VMQ-EVI | .05 | -.01 | .10 | .01 | -.06 |
|  |  | VMQ-IVI | -.06 | -.05 | .04 | .03 | .10 |
|  |  | VMQ-KVI | .05 | .05 | .08 | -.004 | .12 |

*VVIQ* Vividness of Visual Imagery Questionnaire, *SUIS* Spontaneous Use of Imagery Scale, *BAIS-TOT* Bucknell Auditory Imagery Scale - Total, *BAIS-V* Bucknell Auditory Imagery Scale - Vividness, *BAIS-C* Bucknell Auditory Imagery Scale - Control, *IMIS* Involuntary Musical Scale (frequency), *VMIQ-TOT* Vividness of Motor Imagery Questionnaire - Total, *VMIQ-EVI* Vividness of Motor Imagery Questionnaire – External Visual Imagery, *VMIQ-IVI* Vividness of Motor Imagery Questionnaire - Internal Visual Imagery, *VMIQ-KVI* Vividness of Motor Imagery Questionnaire - Kinesthetic Visual Imagery

** p* < .05. ** *p* < .01.

Table 3

Spearman correlations between all mental imagery measures (*n* = 279)

|  | VVIQ | VMIQ-TOT | VMIQ-EVI | VMIQ-IVI | VMIQ-KVI | BAIS-TOT | BAIS-V | BAIS-C | IMIS |
| --- | --- | --- | --- | --- | --- | --- | --- | --- | --- |
| SUIS | .31** | .26** | .25** | .25** | .20** | .37** | .32** | .37** | .10 |
| VVIQ |  | .50** | .43** | .41** | .48** | .49** | .46** | .45** | .00 |
| VMIQ-TOT |  |  | .87** | .89** | .82** | .53** | .53** | .45** | .12 |
| VMIQ-EVI |  |  |  | .67** | .50** | .45** | .46** | .37** | .06 |
| VMIQ-IVI |  |  |  |  | .68** | .50** | .50** | .42** | .14* |
| VMIQ-KVI |  |  |  |  |  | .48** | .46** | .43** | .11 |
| BAIS-TOT |  |  |  |  |  |  | .93** | .92** | .13* |
| BAIS-V |  |  |  |  |  |  |  | .73** | .14* |
| BAIS-C |  |  |  |  |  |  |  |  | .12 |

*SUIS* Spontaneous Use of Imagery Scale, *VVIQ* Vividness of Visual Imagery Questionnaire, *VMIQ-TOT* Vividness of Motor Imagery Questionnaire - Total, *VMIQ-EVI* Vividness of Motor Imagery Questionnaire – External Visual Imagery, *VMIQ-IVI* Vividness of Motor Imagery Questionnaire - Internal Visual Imagery, *VMIQ-KVI* Vividness of Motor Imagery Questionnaire - Kinesthetic Visual Imagery, *BAIS-TOT* Bucknell Auditory Imagery Scale - Total, *BAIS-V* Bucknell Auditory Imagery Scale - Vividness, *BAIS-C* Bucknell Auditory Imagery Scale - Control, *IMIS* Involuntary Musical Scale (frequency),

* *p* < .05*.* ** *p* < .01.
